# A founder deletion in the *TRPM1* gene associated with congenital stationary night blindness and myopia is highly prevalent in Ashkenazi Jews

**DOI:** 10.1038/s41439-019-0076-4

**Published:** 2019-09-12

**Authors:** Yoel Hirsch, David A. Zeevi, Byron L. Lam, Sholem Y. Scher, Rachel Bringer, Bitya Cherki, Cadina C. Cohen, Hagit Muallem, John (Pei-Wen) Chiang, Madhulatha Pantrangi, Josef Ekstein, Martin M. Johansson

**Affiliations:** 1 Dor Yeshorim, The Committee for Prevention of Jewish Genetic Diseases, Brooklyn, NY USA; 2 Dor Yeshorim, The Committee for Prevention of Jewish Genetic Diseases, Jerusalem, Israel; 3 Bascom Palmer Eye Institute, University of Miami Miller School of Medicine, Miami, FL USA; 4 Molecular Vision Laboratory, Hillsboro, OR USA; 5 PreventionGenetics, Marshfield, WI 54449 USA

**Keywords:** Rare variants, Disease genetics

## Abstract

Congenital stationary night blindness (CSNB) is a disease affecting the night vision of individuals. Previous studies identified *TRPM1* as a gene involved in reduced night vision. Homozygous deletion of *TRPM1* was the cause of CSNB in several children in 6 Ashkenazi Jewish families, thereby prompting further investigation of the carrier status within the families as well as in large cohorts of unrelated Ashkenazi and Sephardi individuals. Affected children were tested with a CSNB next-generation (NextGen) sequencing panel. A deletion of *TRPM1* exons 2 through 7 was detected and confirmed by PCR and sequence analysis. A TaqMan-based assay was used to assess the frequency of this deletion in 18266 individuals of Jewish descent. High-throughput amplicon sequencing was performed on 380 samples to determine the putative deletion-flanking founder haplotype. Heterozygous *TRPM1* deletions were found in 2.75% (1/36) of Ashkenazi subjects and in 1.22% (1/82) individuals of mixed Ashkenazi/Sephardic origin. The homozygous deletion frequency in our data was 0.03% (1/4025) and was only found in Ashkenazi Jewish individuals. Homozygous deletion of exons 2–7 in *TRPM1* is a common cause of CSNB and myopia in many Ashkenazi Jewish patients. This deletion is a founder Ashkenazi Jewish deletion.

## Night blindness: Mutation prevalent in Ashkenazi Jews linked to disease

A genetic mutation found in Ashkenazi Jewish population causes an eye disease that leads to poor vision in dim light. Yoel Hirsch and Martin M. Johansson from Dor Yeshorim, together with colleagues determined the genetic etiology of congenital stationary night blindness (CSNB) in children from six Ashkenazi families. Each affected child harbored two mutant versions of *TRPM1*, a gene involved in the transmission of light-elicited signals within the retina of the eye. Notably, all the children had the same large chunk of DNA missing from the gene. The researchers next screened for this genetic deletion in >18,000 individuals of Jewish descent, finding single copies of the mutation in 2.75% of Ashkenazi subjects. The findings should help doctors better diagnose CSNB and care for Jewish patients with eyesight problems.

## Introduction

Congenital stationary night blindness (CSNB) is a disorder of the retina characterized by rod photoreceptor dysfunction or signal transmission defects from photoreceptor to bipolar cells^1^. Symptoms manifest as difficulties in distinguishing objects in low light conditions and can often be accompanied by myopia, hyperopia, reduced visual acuity, strabismus or nystagmus^2^. Inheritance can be autosomal-recessive or X-linked depending on the genes affected^3–5^. A recent observation of CSNB in Appaloosa horses, which exhibited reduced vision in dim light conditions, was linked to significantly reduced expression levels of *TRPM1* mRNA in the retina^6^. *TRPM1* homozygous deletion comprising exons 2–7 has been previously described in a female patient with complete congenital stationary night blindness (cCSNB)^7^. A carrier frequency of 2.1% (1 in 50) for *TRPM1* exon 2–7 deletion has been reported by Chiang et al. in the Ashkenazi Jewish population in an initial screening and PCR method development study^8^. In the same study, 11 individuals carrying a homozygous *TRPM1* exon 2–7 deletion were detected at a frequency of 1.04% (1 in 95), but no clinical information was provided. Here, we describe 14 additional cases of CSNB in 6 Ashkenazi Jewish families due to homozygous *TRPM1* deletions. We also determine with higher accuracy the frequency of *TRPM1* exon 2–7 deletions in Ashkenazi Jewish and non-Jewish populations and confirm previous observation that the exon 2–7 structural variant is indeed a founder Ashkenazi deletion by haplotype analysis. Determining the carrier frequency of this variation within the Jewish population will facilitate future decisions about whether *TRPM1* should be included in Ashkenazi screening panels and will aid genetic diagnosis of patients affected with CSNB.

## Materials and methods

### Ethics statement

Samples from Families 1–5 were obtained with written consent according to the Dor Yeshorim Institutional Review Board guidelines. Samples from Family 6 were obtained in accordance with the University of Miami Institutional Review Board guidelines. Samples included in the TaqMan and haplotype studies were obtained with written patient consent from self-identified individuals enrolled in the carrier testing Dor Yeshorim program to be used for research purposes. Consent was also provided for patient material to be deidentified and used for research purposes to characterize single gene disorders in the Ashkenazi Jewish population^9^. This study was performed in conformance with HIPAA Regulations (March 2015–November 2016).

### Congenital stationary night blindness NextGen panel screening

DNA from the proband in Family 1 was sequenced using a CSNB next-generation sequencing (NGS) panel by a commercial diagnostic laboratory (PreventionGenetics, Marshfield, WI). Genomic DNA from the proband was isolated from peripheral blood sample using the Gentra Puregene Blood Kit (Qiagen, Valencia, CA) according to the manufacturer’s instructions. The CSNB panel includes the following genes: *CABP4, CACNA1F, CACNA2D4, CHM, GNAT1, GPR179, GRM6, LRIT3, NYX, PDE6B, RDH5, RHO, RPE65, SAG, SLC24A1* and *TRPM1*. For this NGS test, the full coding regions plus ~20 base pairs (bp) of noncoding DNA flanking each exon were sequenced for the genes indicated. Sequencing was accomplished by capturing specific regions with an optimized solution-based hybridization kit, followed by massively parallel sequencing of the captured DNA fragments. Additional Sanger sequencing was performed for any regions not captured or with an insufficient number of sequence reads.

### Deletion confirmation for other family members

**Fig. 1:**
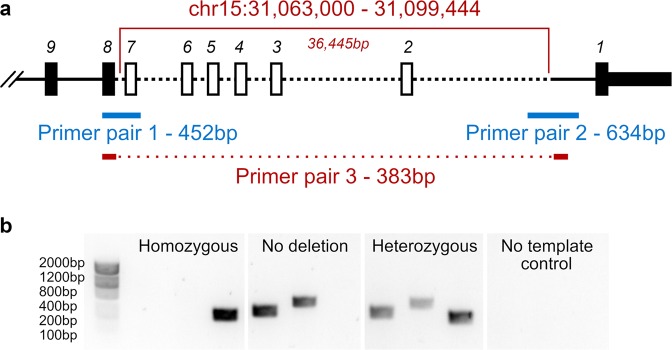
Verification of *TRPM1* deletion by PCR. **a** Schematic representation of the deleted region from exons 2–7 and the indication of regions amplified by PCR primer pairs in blue and red. Boxes represent exons, and dashed lines represent deleted regions (Table S1). **b** Three examples of copy number detection of *TRPM1* exon 2–7 deletion by PCR amplification. The first box shows three PCRs in one individual with a homozygous deletion. Only primer pair 3 shows amplification. The second box shows an individual without a *TRPM1* exon 2–7 deletion where primer pairs 1 and 2 amplify. The third box exhibits amplification of all three primer pairs, indicating a heterozygous deletion. The fourth box shows no template control

Genomic DNA from members of Family 1 and Family 2 was subjected to PCR amplification and direct sequencing. PCR amplification was performed in three separate reactions (Fig. 1 and Table S1). Each reaction mix consisted of 1 µl of gDNA (20 ng), 0.1 µl of Kapa2G Fast Kit DNA Polymerase (Kapa Biosystems), 0.5 µl of dNTP (10 nM), 5 µl of 5x Reaction Buffer, 1 µl of Primer PCR-F (5 µM), 1 µl of Primer PCR-R (5 µM) and 11.4 µl of water in a total volume of 20 µl. The PCR cycling conditions consisted of an initial denaturation at 95 °C for 3 min, followed by two different cycling conditions: the first one consisted of 5 cycles of amplification; 95 °C for 10 s, 64 °C for 15 s, and 72 °C for 15 s and the second one consisted of 35 cycles of amplification; 95 °C for 10 s, 61 °C for 15 s, and 72 °C for 15 s. The final extension phase was performed for 2 min. PCR products were run on a 1.5% agarose gel (agarose 1× TAE buffer). To ensure the specificity of the amplified regions, the PCR product was enzymatically purified using an in-house ExoSAP protocol, and end sequencing was performed in-house using BigDye Terminator Cycle Sequencing. PCR was performed by GENEWIZ according to standard operating procedures, and primer extension sequencing was performed by GENEWIZ, Inc. (South Plainfield, NJ) using Applied Biosystems BigDye version 3.1. The reactions were then run on Applied Biosystem’s 3730xl DNA Analyzer.

### PCR confirmation by duplex PCR

Probands from Families 3, 4, and 5 were identified through TaqMan assay screening and confirmed by a duplex PCR method. The additional homozygotes in the abovementioned families were only genotyped by duplex PCR. PCR was performed using the KAPA2G PCR Kit (Kapa Biosystems) and was run on the program 94 °C for 60 s followed by 35 cycles of 30 s at 94 °C, 30 s at 55 °C and 40 s at 72 °C. Gel electrophoresis was performed on the QIAxcel Advanced gel electrophoresis system (Qiagen). PCR primers were designed using the Primer3 program (http://bioinfo.ut.ee/primer3-0.4.0/primer3/).

### Vision Panel screening

Direct testing for mutations in the genes of the Molecular Vision Laboratory (MVL) Vision Panel v2 (https://www.molecularvisionlab.com/mvl-vision-panel/) was performed by target enrichment (capture) and NGS for the proband in Family 6. MVL Vision Panel v2 consists of 581 genes with an average coverage of ~700 reads and at least 30× coverage in >96% of the panel. Targeted regions cover all exons, exon-intron boundaries, and relevant, deep-intronic regions. Identified mutations and novel variations were confirmed by Sanger sequencing. All exons and exon/intron boundaries were sequenced. Exon 1 is defined as the exon having the start codon ATG. Codon 1 corresponds to the start ATG and nucleotide 1 to the A. Alignment to GRCh37 was performed using NextGENe, and variants were called in Geneticists Assistant (SoftGenetics, State Collage, Pennsylvania, USA) according to American College of Medical Genetics (ACMG) guidelines^10^. Variants with a dbSNP allele frequency >20% or previously identified as false positives were excluded.

### *TRPM1* deletion screening by TaqMan

Genomic DNA from 12075 individuals of Ashkenazi Jewish origin, 3238 of Sephardi origin and 2953 of mixed Ashkenazi and Sephardi Jewish ancestry were screened for the *TRPM1* exon 2–7 deletion according to a previously described high-throughput TaqMan allelic discrimination genotyping method on the Fluidigm platform^11^. The custom TaqMan genotyping assay for the *TRPM1* deletion (with proprietary primer and probe sequences) was purchased from QuantaBio (Beverly, MA, USA). Briefly, the assay was designed as a multiplex of one primer pair and HEX-labeled probe targeting the wild-type *TRPM1* sequence (deleted in carriers) and another primer pair and 6-FAM-labeled probe targeting the deletion breakpoint (which is not present in homozygous wild-type individuals). Sanger sequencing information from the homozygous deletion carrier in Family 1 was used to design the deletion breakpoint-spanning portion of the custom TaqMan assay.

### Ethnic origin definition of study participants

Participants in the TaqMan-based copy number variation (CNV) screening provided information regarding their maternal and paternal ethnicity. Self-identification has been previously shown to be accurate^12^. For the analysis by country of origin, out of 18266 individuals investigated, 2486 were retained after meeting the first criteria of all four (two maternal and two paternal) ancestors being from same country of origin, or the second criteria where only one maternal and one paternal ancestor was provided, and both originated from the same country. Individuals who had Israel or United States in their ancestry were removed from analysis, since the Jews residing in these countries are often of mixed Ashkenazi Jewish origin. Finally, 1966 individuals were retained for frequency by country analysis.

### SNP and Copy Number array analysis

In total, we investigated 835 publicly available intensity data files from Genome-Wide Human SNP array 6.0 (Affymetrix, Santa Clara, CA, USA), out of which we retained 746 after quality control. The Ashkenazi Jewish group consisted of 385 genomes, and the non-Jewish group consisted of 361 genomes from various populations (Table S2) that were determined by original studies. Additionally, one proband from Families 1 and 2 with a known homozygous deletion of *TRPM1* was subjected to genotyping on the Genome-Wide Human SNP Array 6.0. Analysis of intensity data files was performed on Affymetrix Genotyping Console Software 4.3.840 (GTC). Copy number/LOH analysis was performed using Regional GC Correction, and all samples were normalized against the GenomeWideSNP_6.hapmap270.na33.r1.a5.ref reference samples. GenomeWideSNP_6.na33.annot.db was used as the annotation reference file. For segment reporting, the following settings were used: minimum number of markers per segment = 5, minimum genomic size of a segment (kbp) = 10, including segments that overlap with known CNV = 100. Samples with a median of the absolute values of all pairwise differences (MAPD) value above 0.35 were excluded from further analysis, resulting in 387 Ashkenazi and 361 gentile samples being analyzed. The Log2Ratio signal was also analyzed by the CNV.BEAST algorithm which uses a robust backward elimination regression approach^13^. Analysis was performed on standard settings.

### Haplotype determination by high-throughput amplicon sequencing

SNP array analysis of two homozygous deletion carriers identified a long shared region of homozygosity flanking the *TRPM1* gene. Eleven SNPs were selected from this data (9 SNPs within the homozygous region and 1 additional flanking SNP just upstream and downstream of the region) for further genotyping of 380 additional samples (Table S3). Briefly, a multiplex PCR assay was used to target 11 SNPs in barcode indexed samples by high-throughput sequencing on a MiSeq instrument (Illumina, San Diego, California, USA) (Table S4). Resultant sequencing reads were aligned to the reference genome (hg38), and relevant SNPs were genotyped using the “emit_all_sites” flag within the GATK UnifiedGenotyper package (Broad Institute).

### Statistical analysis

A two-sided Fisher´s exact test on the observations from the TaqMan and haplotype studies was performed using R language and statistical environment (version 3.5.1)^14^.

## Results

### Family 1

**Fig. 2:**
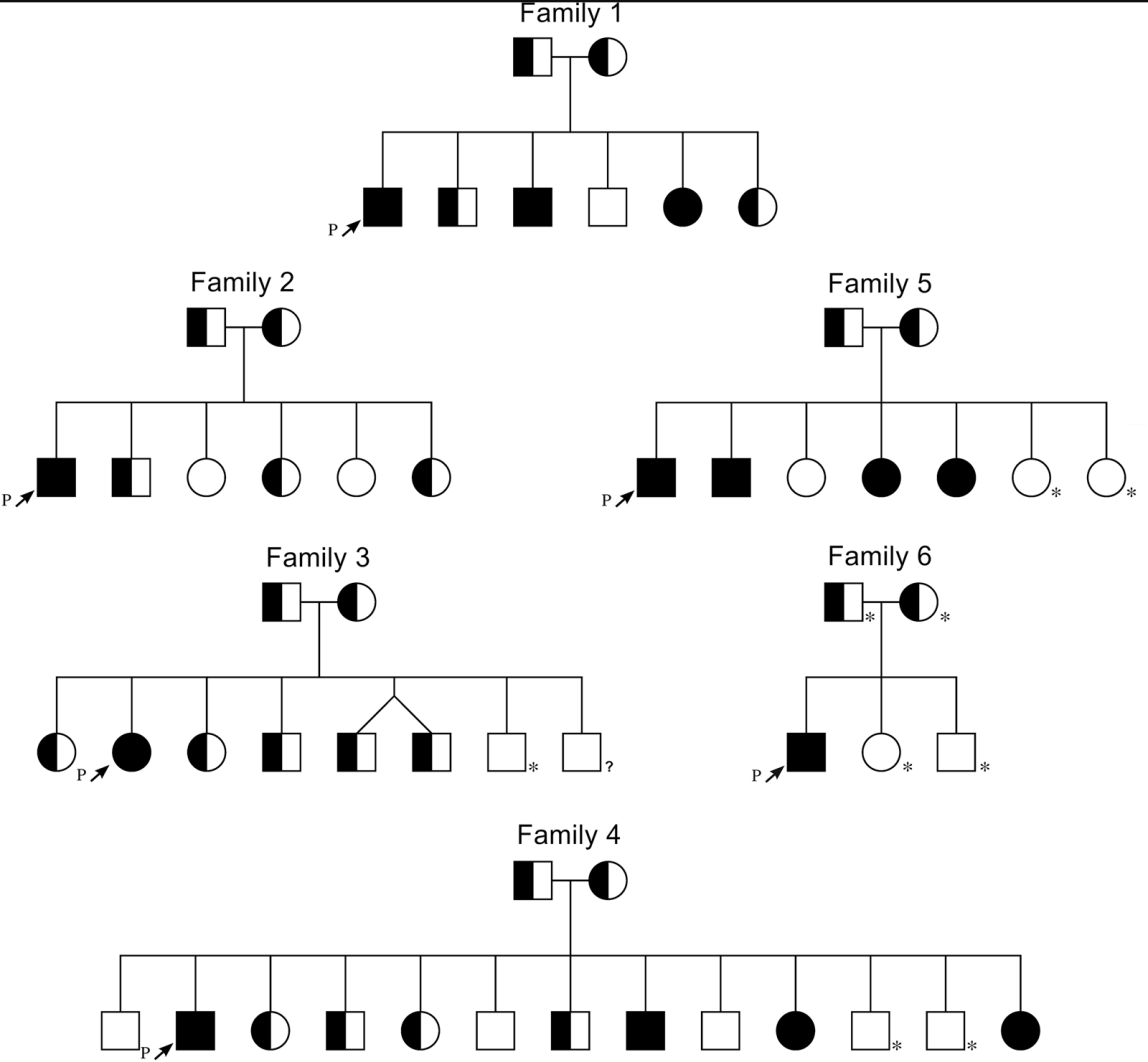
Pedigree of *TRPM1* families. Segregation pedigrees for *TRPM1* exon 2–7 deletion. Filled symbols = affected, half filled = heterozygous carrier, unfilled = noncarrier, unfilled with star = not clinically affected and not tested, * = not genetically tested, ? = symptoms without genetic testing

Proband 1 was the first of 6 children to healthy unrelated Ashkenazi Jewish parents of Hungarian origin. At age 3, he was diagnosed with myopia, which progressed to −13 diopter bilaterally by age 18 years with the best-corrected visual acuity of 20/30 in each eye (Table 1). He was noted to have tilted myopic optic nerve heads and myopic retinal changes, and his myopia subsequently stabilized. At night in dim light, his vision is mostly diminished. The proband’s affected sister was diagnosed with myopia a few months after birth and started to wear glasses at the age of 2 years. Her myopia has progressed as in proband 1. By age 14 years, she was −7 diopter with 2 diopter of astigmatism bilaterally with 20/30 in each eye. Additionally, she also exhibits CSNB similar to proband 1 (Fig. 2). The proband’s affected brother was diagnosed with myopia shortly after birth and exhibits CSNB symptoms.

**Table 1:** Clinical information of probands with homozygous *TRPM1* deletion

	Affected individual	Gender	Age of diagnosis	CSNB	Myopia	Strabismus	Nystagmus
Family 1	1*	Male	3 years	Yes	Yes	Esophoria	No
	2	Female	5 months	Yes	Yes	Esophoria	No
	3	Male	5 months	Yes	Yes	Esophoria	No
Family 2	4*	Male	10 months	Yes	Yes	Mild	No
Family 3	5*	Female	18 months	Yes	Yes	Esophoria	No
Family 4	6*	Male	6 months	Yes	Yes	Exotropia	No
	7	Male	Na	icCSNB^a^	Yes	Exotropia	No
	8	Female	Na	Yes	Yes	Exotropia	No
	9	Female	Na	Yes	Yes	Exotropia	No
Family 5	10*	Male	4 months	icCSNB^a^	Yes	Exotropia	No
	11	Male	6–12 months	icCSNB^a^	Yes	Exotropia	No
	12	Female	6–12 months	icCSNB^a^	Yes	Exotropia	No
	13	Female	6–12 months	icCSNB^a^	Yes	Exotropia	No
Family 6	14*	Male	4 months	Yes	Yes	Esotropia	Yes

*Proband

^a^Incomplete Congenital Stationary Night Blindness

*Na* Not available

**Fig. 3:**
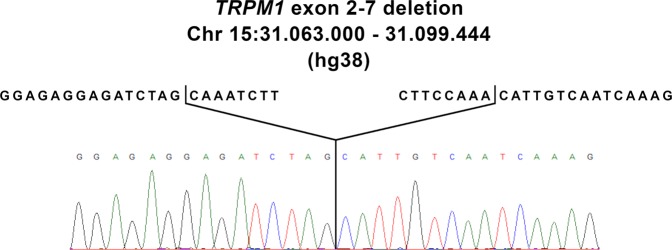
*TRPM1* breakpoint determination. The upper sequence shows the nondeleted DNA reference sequence, while the lower sequence shows the read-out from Sanger Sequencing, indicating the breakpoint location

In our attempt to find the cause of the observed CSNB symptoms in the proband, we tested DNA using a CSNB NGS sequencing panel, which indicated dropped read coverage for exons 2 through 7 in the *TRPM1* gene. A deletion of 36,455 bp affecting exons 2–7 was confirmed by PCR and Sanger sequencing on chromosome 15 at genomic location 31,063,000–31,099,444 (hg38). This deletion corresponds to (NM_001252020.1 (*TRPM1*): c.55-22460_1016 + 118del) (Fig. 3) and is identical to what was previously described^7^.

To confirm the pathogenicity of the abovementioned deletion, we performed segregation analysis on the proband’s parents and siblings and found that the parents and two unaffected siblings were heterozygous carriers, one sibling was homozygous wild type, and two additional affected siblings described above were homozygous for the *TRPM1* deletion.

### Family 2

We also investigated another Ashkenazi Jewish family of Hungarian descent related to Family 1, with one child exhibiting CSNB symptoms by using the PCR and Sanger sequencing methods as described above. The same method was used for Families 2–5. We found a homozygous deletion in the symptomatic child. The parents were heterozygous carriers, and unaffected siblings were either heterozygous or did not carry the deletion.

### Family 3

The parents are not related and of Ashkenazi descent. The father shares Ukrainian and Romanian Jewish origins, and the mother is of Polish Jewish ancestry. Proband 5 is a female who is the only one out of 8 children affected by CSNB and who carries the *TRPM1* exon 2–7 deletion. She was born after an uneventful pregnancy and was diagnosed at 18 months of age with myopia requiring a prescription of −8.00 diopter. She experiences night blindness and suffered from strabismus, which was treated surgically. One male sibling was diagnosed with myopia at age 4 but did not experience CSNB and was not subjected to genetic testing.

### Family 4

The parents are of full Ashkenazi descent and not related. The father is from Russia, and the mother is of Polish origin. Four out of 13 children have vision impairment and exhibit a homozygous deletion. The affected proband 6 is a male who is currently age 34. At the age of 6 months, he was diagnosed with strabismus and shortly thereafter exhibited severe myopia. At age 7, he underwent a surgical procedure to correct the strabismus that did not correct the exotropia. He suffers from severe night blindness. Affected individual 7 is also a male with mild strabismus that was successfully treated by vision therapy. He has mild CSNB, myopia and strabismus. Affected individual 8 is a female currently at the age of 18 years with severe strabismus. She underwent three surgical procedures during childhood to correct strabismus. She also has CSNB and myopia. Affected individual 9 is an 8-year-old female with exotropia and is currently treated with vision therapy. She exhibits CSNB with myopia.

### Family 5

The parents are nonconsanguineous Ashkenazi Jews of Lithuanian and Russian descent, with four children out of 7 exhibiting exotropia, myopia and CSNB caused by the homozygous *TRPM1* exon 2–7 deletion. Two siblings are males aged 21 and 19 years, and two are females aged 14 and 12 years. Symptoms of strabismus followed shortly after birth and were successfully treated with vision therapy without requiring any surgical procedure.

### Family 6

The parents are of Ashkenazi Jewish descent. The father’s Jewish parents are from Moldova and Ukraine, and the mother’s Jewish parents are from Ukraine and Israel. There is no evidence of known consanguinity for at least 5 generations. One of the 3 children is affected and has congenital nystagmus, early-onset esotropia with disassociated vertical deviation, myopia, and CSNB. Strabismus surgeries were performed at ages 18 and 23. At age 30, the patient’s visual acuity was 20/30 in each eye. The affected child is homozygous for the *TRPM1* exon 2–7 deletion, which was detected using the MVL Vision Panel v2 method as described in the Methods section.

### *TRPM1* deletion frequency

To establish the frequency of *TRPM1* deletions in Jewish populations, 18,266 individuals were screened using a high-throughput TaqMan allelic discrimination genotyping method. In 12,075 Ashkenazi individuals, 332 had heterozygous deletions, resulting in a frequency of 2.75% or 1 in 36 carrier frequency (Table 2). In 3238 individuals with Sephardi Jewish descent, we found no deletion carriers. Finally, in 2953 subjects of mixed Ashkenazi and Sephardi origin, we found 36 heterozygous carriers, resulting in a frequency of 1.22% or 1 in 82. Homozygous deletions were identified in 3 out of 12,075 Ashkenazi individuals, resulting in a predicted disease frequency of 0.03% or 1 in 4025 if the condition is fully penetrant.

**Table 2:** Frequency of *TRPM1* exon 2–7 deletions detected by TaqMan allelic discrimination genotyping

Population	Non-carrier Individuals	Heterozygous CNV	Frequency heterozygous
Ashkenazi	11743	332	2.75% (1/36)
Sephardi	3238	0	0%
Mixed	2917	36	1.22% (1/82)

### SNP array results

To further assess the frequency of the deletion in the Ashkenazi population, we compared publicly available intensity data files from Ashkenazi Jewish individuals against non-Jews. In the genomes of 385 Ashkenazi Jewish individuals, we detected 14 *TRPM1* heterozygous deletions (Fig. S1) at a frequency of 3.64% or 1 in 28. Size ranged from 15 to 41 kb with an average of 29.1 kb (Table S5). All *TRPM1* deletions were heterozygous. No *TRPM1* exon 2–7 deletions were observed in 361 non-Jewish individuals tested. All 14 deletions detected by the Canary Algorithm above were also detected as heterozygous CNVs by the CNV.BEAST algorithm (Table S6). Fisher’s exact test indicated a significant overrepresentation of carriers observed in Ashkenazi compared to non-Jewish SNP array samples (*p* = 0.0001). When contrasting the frequencies of carriers in SNP array data to TaqMan Ashkenazi data, Fisher’s exact test did not detect any significant difference between these two groups (*p* = 0.34).

### *TRPM1* deletion haplotype confirmation

To determine whether the *TRPM1* deletion is a founder variant, we performed SNP arrays on two probands from Families 1 and 2, both homozygous for the deletion. This preliminary test identified a relatively long region of homozygosity flanking the *TRPM1* gene, which was 1.9 Mb in size. Using this region of homozygosity as our “working” founder haplotype, we selected 9 SNPs within the region (and two other control SNPs outside the region of homozygosity) for genotyping of the 265 heterozygous deletion carriers in addition to 4 more unrelated homozygous deletion carriers and other noncarrier controls (Table S3). For high-throughput genotyping of the 11 SNPs, we performed custom amplicon sequencing of 380 samples altogether (Table S4).

**Fig. 4:**
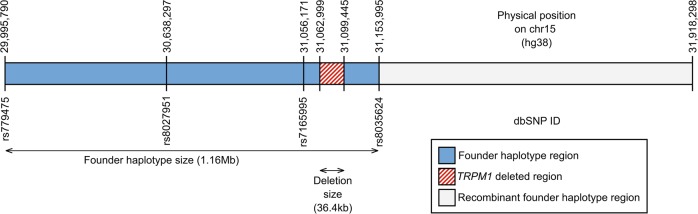
Illustration of the AJ founder haplotype relative to the *TRPM1* deleted region on chromosome 15. The founder haplotype was detected in all *TRPM1* deletion carriers. The recombinant founder haplotype was present in most but not all *TRPM1* deletion carriers. The founder haplotype is in blue, the *TRPM1* deleted region is indicated in red stripes, and the recombinant founder haplotype region is in gray

The four homozygous deletion carriers shared the same gene-flanking haplotype on 5 out of 11 deletion-flanking SNPs genotyped by sequencing. This haplotype of 1.16 Mb (Table S4, Fig. 4) is shared among all deletion carriers from Ashkenazi and mixed Ashkenazi groups. Ashkenazi noncarriers and non-Ashkenazi noncarriers exhibit a more equal distribution of consistent vs. inconsistent genotypes associated with the deletion haplotype (Table S7). Accordingly, Fisher’s exact test indicates that the founder Ashkenazi carriers are genetically distinct from the Ashkenazi noncarriers and non-Ashkenazi noncarriers (*p* < 2.2e^−16^) for the haplotype investigated. Furthermore, the genetic distinctiveness between mixed Ashkenazi carriers and non-Ashkenazi noncarriers is also significant (*p* = 1.29e^−7^). Additionally, all 14 carriers and the two affected children detected in the SNP array investigation exhibit identical haplotype as the Ashkenazi Jewish carriers and affected individuals in the amplicon sequencing study.

### Geographical frequencies of carriers in TaqMan assay data

Carrier rates in samples from the TaqMan assay study show that the *TRPM1* exon 2–7 deletion frequency is highest among Ashkenazi Jews with historical background in Romania (7.6%), followed by Ukraine (5.3%), Poland (4.2%), Germany and Lithuania (3.9%), Russia (3.0%) and Hungary (2.6%) (Fig. S2). Fisher’s exact test does not indicate significant overrepresentation of carrier frequency in any of the groups (*p* = 0.23).

## Discussion

We demonstrated a homozygous deletion in exons 2–7 of *TRPM1* in 6 Ashkenazi Jewish families with early-onset myopia and CSNB. Associated strabismus and nystagmus are of variable penetrance. Strabismus has in several cases been successfully treated by vision therapy or surgery. Notably, the clinical findings of our patients are consistent with a recently described large cohort of *TRPM1*-associated CSNB in children^15^.

According to our study, the *TRPM1* exons 2–7 deletion is exclusively found in the Jewish population and is a founder mutation. It has been reported in two other studies to date, and the affected individuals were also of Ashkenazi Jewish origin^7,8^.

The high carrier frequency identified in our study can be explained by the fact that the phenotypes associated with *TRPM1* deletion are not severe enough to frequently prompt genetic assessment. The most frequent phenotype is myopia, which is common in the general population^16^. Although the onset of myopia in children with homozygous *TRPM1* deletion manifests earlier in comparison with myopia caused by nongenetic factors^17^, its progression stabilizes over time. Similarly, strabismus is in many cases corrected by vision therapy or surgical correction. Nyctalopia may be underreported because the environment is often illuminated at night in cities.

The SNP array investigation supports the significant overrepresentation of the *TRPM1* deletion in Ashkenazi Jewish individuals compared to four contrasting populations. The higher heterozygous frequency of 3.64% could be explained by the limited number of samples investigated compared to the TaqMan genotyped cohort. It is important to note that the *TRPM1* heterozygous deletions detected in the SNP microarray data differ in terms of exons affected. This is because not all exons within *TRPM1* have a SNP within the exon, limiting the ability to accurately define the deletion size. In this case, exons 7–10 are not represented by any probes that render the exact deletion breakpoint determination by Affymetrix Genome-Wide Human SNP Array 6.0 impossible. However, the presence of a single exclusive *TRPM1* deletion-flanking haplotype indicates that these deletions are all identical. The probability that detected CNVs are false positive is low since we tested signal intensity by two detection algorithms that both detected the deletions. Overall, the results from the SNP array investigation reflected the frequency of heterozygous deletions in the Ashkenazi group of the TaqMan study and exhibited the exclusivity of *TRPM1* exon 2–7 deletion to the Jewish population.

A study from 1993 based on data from 341,175 military recruits in Israel reports CSNB at a substantially lower frequency of 1 in 10,661 compared to the prevalence in our study of 1 in 4025^18^. One explanation for the observed discrepancy could be the fact that the military recruits were not screened by a genetic test but by a medical history questionnaire. This allowed individuals with homozygous deletion and a mild phenotype of CSNB to pass undetected. Another plausible explanation could be that the ancestry of the recruits differed substantially from the individuals tested at Dor Yeshorim, which to a larger extent consists of Ashkenazi Jewish individuals.

As opposed to other studies, the observed northeastern-southcentral cline for certain Ashkenazi-specific pathogenic variants was not detected in our analysis by country^19–23^. This observation is also reinforced by self-reported ancestry of the parents in the six families that are distributed between Polish, Hungarian, Lithuanian, Ukrainian and Russian Jewish ancestry.

The fact that the affected children and all carriers exhibit an identical haplotype proves our hypothesis that *TRPM1* exon 2–7 deletion is an Ashkenazi Jewish founder event. The absence of several haplotypes excludes the possibility that this CNV occurred as a hot spot event but rather resulted from a single founder event that is transmitted by classic meiosis. This excludes the probability that this deletion could vary in size or encompass nearby genes causing a more severe phenotype with additional characteristics.

Although the carrier frequency exceeds one of the defined criteria of American College of Medical Genetics for carrier screening, namely, of >1% heterozygous variant prevalence in the Ashkenazi Jewish population, the second criterion is not met because CSNB and myopia does not pose a significant morbidity risk^24^.

We demonstrated with full certainty that the *TRPM1* deletion of exons 2–7 is a founder Ashkenazi deletion. This information will assist physicians caring for patients with juvenile myopia and CSNB to efficiently determine the underlying genetic etiology of the disorders.

## Supplementary information

Supplementary figure 1.

Supplementary figure 2.

Supplementary table 1.

Supplementary table 2.

Supplementary table 3.

Supplementary table 4.

Supplementary table 5.

Supplementary table 6.

Supplementary table 7.

Supplementary figs. legend.
