# Supplementary figures and images for: A founder deletion in the TRPM1 gene associated with congenital stationary night blindness and myopia is highly prevalent in Ashkenazi Jews
Source: Hum Genome Var. 2019 Sep 12;6:45. doi: 10.1038/s41439-019-0076-4 (PMC6804618; doi:10.1038/s41439-019-0076-4)

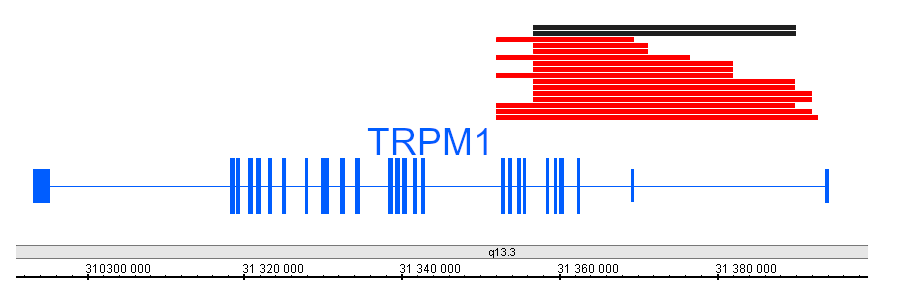

Supplement: Supplementary file 1 — Supplementary figure 1. [file 41439_2019_76_MOESM1_ESM.gif]

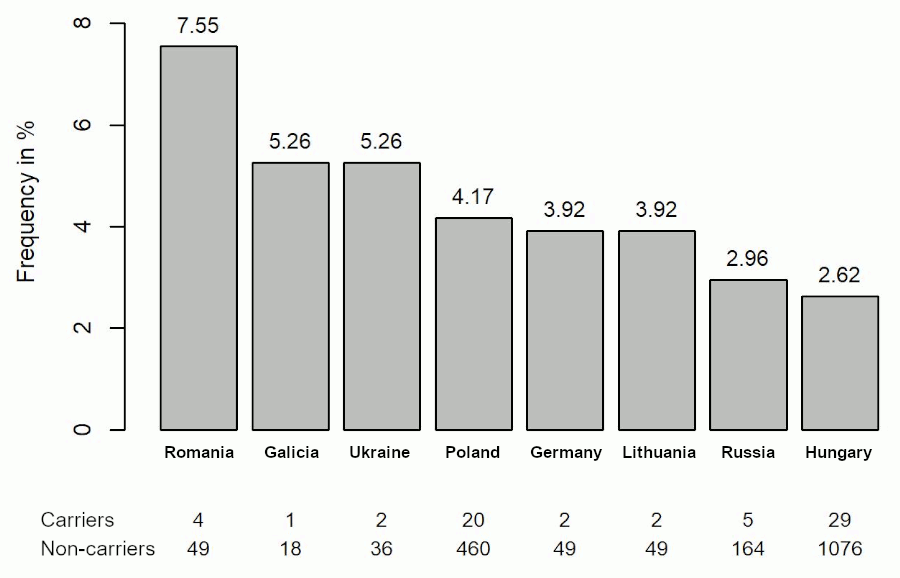

Supplement: Supplementary file 2 — Supplementary figure 2. [file 41439_2019_76_MOESM2_ESM.gif]
