# Supplementary material for: A founder deletion in the TRPM1 gene associated with congenital stationary night blindness and myopia is highly prevalent in Ashkenazi Jews
Source: Hum Genome Var. 2019 Sep 12;6:45. doi: 10.1038/s41439-019-0076-4 (PMC6804618; doi:10.1038/s41439-019-0076-4)
Supplement: Supplementary file 3 — Supplementary table 1. [file 41439_2019_76_MOESM3_ESM.docx]

Supplementary Table 1. Primer sequences and amplification information for *TRPM1* exon 2-7 deletion confirmation by PCR

| PCR | F Primer Name | F Primer Sequence | R Primer Name | R Primer Sequence | Information |
| --- | --- | --- | --- | --- | --- |
| PCR 1 | TRPM1-5’F | 5’-GCTCTGTAAAGCTGATAAGC-3’ | TRPM1-5’R | 5’-GAGTCTAACCTCAGAGCTTG-3’ | Amplification if 5’ region is present |
| PCR 2 | TRPM1-3’F | 5’-AATTCCTTCCAGCCAGA-3’ | TRPM1-3’R | 5’-CTTTGCTGTGGGAGGATATC-3’ | Amplification if 3’ region is present |
| PCR 3 | TRPM1-5’F | 5’-GCTCTGTAAAGCTGATAAGC-3’ | TRPM1-5’R | 5’-CTTTGCTGTGGGAGGATATC-3’ | Amplification if *TRPM1* exon 2-7 is missing |
