# Supplementary material for: A founder deletion in the TRPM1 gene associated with congenital stationary night blindness and myopia is highly prevalent in Ashkenazi Jews
Source: Hum Genome Var. 2019 Sep 12;6:45. doi: 10.1038/s41439-019-0076-4 (PMC6804618; doi:10.1038/s41439-019-0076-4)
Supplement: Supplementary file 4 — Supplementary table 2. [file 41439_2019_76_MOESM4_ESM.docx]

Supplementary Table 2. Numbers and origins of CEL files included in *TRPM1* screening

| Ethnicity | Study ID | CEL Files | CEL files retained after QC |
| --- | --- | --- | --- |
| Ashkenazi Jewish | GSE23201 | 400 | 385 |
| Affected proband 2 and 4 | Dor Yeshorim | 2 | 2 |
| Bolivian | GSE29851 | 47 | 19 |
| Kashmiri | GSE85594 | 47 | 38 |
| Chinese | GSE80481 | 155 | 149 |
| Chinese | GSE53790 | 64 | 55 |
| Chinese | GSE1833 | 33 | 11 |
| Yoruba | HapMap3 | 89 | 89 |

QC – Quality Control
