# Supplementary material for: A founder deletion in the TRPM1 gene associated with congenital stationary night blindness and myopia is highly prevalent in Ashkenazi Jews
Source: Hum Genome Var. 2019 Sep 12;6:45. doi: 10.1038/s41439-019-0076-4 (PMC6804618; doi:10.1038/s41439-019-0076-4)
Supplement: Supplementary file 5 — Supplementary table 3. [file 41439_2019_76_MOESM5_ESM.docx]

Supplementary Table 3. Ancestry group and number of samples included in the haplotype determination

| Haplotype determination cohort | Number of individuals |
| --- | --- |
| Ashkenazi Jewish homozygous affected | 4 |
| Ashkenazi Jewish carriers | 237 |
| Mixed Ashkenazi Jewish carriers | 28 |
| Ashkenazi Jewish non-carriers | 56 |
| Non-Ashkenazi Jewish non-carriers | 55 |
| Total | 380 |
