# Supplementary material for: A founder deletion in the TRPM1 gene associated with congenital stationary night blindness and myopia is highly prevalent in Ashkenazi Jews
Source: Hum Genome Var. 2019 Sep 12;6:45. doi: 10.1038/s41439-019-0076-4 (PMC6804618; doi:10.1038/s41439-019-0076-4)
Supplement: Supplementary file 6 — Supplementary table 4. [file 41439_2019_76_MOESM6_ESM.docx]

Supplementary Table 4. Evidence for founder allelic inheritance of the *TRPM1* deletion in AJ carriers

| SNP rs ID | Reference allele | Alternate allele | Homozygous deletion carrier haplotype*^c^* | chr15 position hg38 | Carrier AJ Samples (n=237) Founder allele fraction*^d^* | Carrier Mixed AJ Samples (n=28) Founder allele fraction*^d^* | Non-carrier AJ Samples (n=56) Founder allele fraction*^d^* | Non-carrier Non-AJ samples (n=55) Founder allele fraction*^d^* |
| --- | --- | --- | --- | --- | --- | --- | --- | --- |
| rs11637663 | G | A | NA | 29,995,625 | NA | NA | NA | NA |
| rs4779475 | G | C | G | 29,995,790 | 1.00 | 1.00 | 0.95 | 1.00 |
| rs8027951 | G | T | T | 30,638,297 | 1.00 | 1.00 | 0.70 | 0.80 |
| rs7182907*^a^* | C | T | C | 31,005,662 | 1.00 | 1.00 | 1.00 | 1.00 |
| rs7165995*^b^* | G | A | A | 31,056,171 | 1.00 | 1.00 | 0.86 | 0.91 |
| rs4779824*^b^* | T | C | C | 31,112,091 | 1.00 | 1.00 | 0.79 | 0.87 |
| rs8035624 | A | C | A | 31,153,995 | 1.00 | 1.00 | 0.91 | 0.85 |
| rs8035633 | G | A | A | 31,407,819 | 0.97 | 1.00 | 0.86 | 0.85 |
| rs7178914 | C | T | C | 31,616,932 | 0.95 | 1.00 | 0.91 | 0.96 |
| rs17228459 | C | T | T | 31,906,136 | 0.92 | 1.00 | 0.55 | 0.87 |
| rs1996171 | A | G | NA | 31,918,298 | NA | NA | NA | NA |

*^a^* This SNP was not informative in this study; it was homozygous reference in all samples

*^b^* Deletion flanking SNPs

*^c^* These alleles were homozygous in the microarray data from 2 of the homozygous deletion carriers in the study (note that not all homozygous deletion carriers in the study were homozygous for every allele in this haplotype)

*^d^* Fraction of samples with at least 1 founder allele nucleotide

NA, not applicable

AJ, Ashkenazi Jewish

High throughput sequencing was used to genotype all SNPs in the table on samples from each of the four study cohorts (carrier AJ; carrier mixed AJ; non-carrier AJ; non-carrier non-AJ). The table summarizes key features of each SNP and genotyping outcomes according to founder allele fraction of each SNP in the samples from each cohort.

Shaded rows highlight alleles that were consistently present in all deletion carriers but not necessarily in non-deletion carriers. This is the assumed deletion-linked founder haplotype.
