# Supplementary material for: A founder deletion in the TRPM1 gene associated with congenital stationary night blindness and myopia is highly prevalent in Ashkenazi Jews
Source: Hum Genome Var. 2019 Sep 12;6:45. doi: 10.1038/s41439-019-0076-4 (PMC6804618; doi:10.1038/s41439-019-0076-4)
Supplement: Supplementary file 7 — Supplementary table 5. [file 41439_2019_76_MOESM7_ESM.docx]

Supplementary Table 5. Heterozygous *TRPM1* deletions detected by Canary algorithm

| Sample ID | Copy Number | Size (kb) | Chromosomal Position | Start Marker | End Marker |
| --- | --- | --- | --- | --- | --- |
| GSM574713_MS4A2 | 1 | 41 | Chr 15:31352063-31392887 | CN_689552 | CN_689566 |
| GSM574720_MS4B10 | 1 | 40 | Chr 15:31352063-31392141 | CN_689552 | SNP_A-8477902 |
| GSM574609_MS2G12_MSSM | 1 | 38 | Chr 15:31352063-31389948 | CN_689552 | CN_689565 |
| GSM574803_MS5B4 | 1 | 35 | Chr 15:31356726-31392141 | SNP_A-8466793 | SNP_A-8477902 |
| GSM574877_MS7B12 | 1 | 35 | Chr 15:31356726-31392141 | SNP_A-8466793 | SNP_A-8477902 |
| GSM574623_MS2H7 | 1 | 33 | Chr 15:31356726-31389948 | SNP_A-8466793 | CN_689565 |
| GSM574864_MS7A1 | 1 | 33 | Chr 15:31356726-31389948 | SNP_A-8466793 | CN_689565 |
| GSM574643_MS3B5 | 1 | 30 | Chr 15:31352063-31381989 | CN_689552 | SNP_A-8644108 |
| GSM574776_MS4G6 | 1 | 25 | Chr 15:31356726-31381989 | SNP_A-8466793 | SNP_A-8644108 |
| GSM574938_MS7G6 | 1 | 25 | Chr 15:31352063-31376696 | CN_689552 | SNP_A-8520220 |
| GSM574947_MU1_AP2G12 | 1 | 25 | Chr 15:31356726-31381989 | SNP_A-8466793 | SNP_A-8644108 |
| GSM574940_MS7H1 | 1 | 17 | Chr 15:31352063-31369498 | CN_689552 | SNP_A-1864629 |
| GSM574699_MS3G7 | 1 | 15 | Chr 15:31356726-31371281 | SNP_A-8466793 | SNP_A-8716774 |
| GSM574802_MS5B3 | 1 | 15 | Chr 15:31356726-31371281 | SNP_A-8466793 | SNP_A-8716774 |
