# Supplementary material for: A founder deletion in the TRPM1 gene associated with congenital stationary night blindness and myopia is highly prevalent in Ashkenazi Jews
Source: Hum Genome Var. 2019 Sep 12;6:45. doi: 10.1038/s41439-019-0076-4 (PMC6804618; doi:10.1038/s41439-019-0076-4)
Supplement: Supplementary file 8 — Supplementary table 6. [file 41439_2019_76_MOESM8_ESM.docx]

Supplementary Table 6. Heterozygous *TRPM1* deletions detected by CNV.BEAST algorithm

| Sample | Type | Size (kb) | Start | End | Height | Score | Start probe | End probe |
| --- | --- | --- | --- | --- | --- | --- | --- | --- |
| GSM574713_MS4A2 | 1 | 26.7 | 31344542 | 31371281 | -0.30522128 | 1.72659232 | CN_689549 | SNP_A-8716774 |
| GSM574720_MS4B10 | 1 | 25.0 | 31344542 | 31369498 | -0.40215361 | 2.16566347 | CN_689549 | SNP_A-1864629 |
| GSM574609_MS2G12_MSSM | 1 | 40.8 | 31352063 | 31392887 | -0.37106040 | 2.28736993 | CN_689552 | CN_689566 |
| GSM574803_MS5B4 | 1 | 47.6 | 31344542 | 31392141 | -0.28434782 | 1.84278447 | CN_689549 | SNP_A-8477902 |
| GSM574877_MS7B12 | 1 | 33.4 | 31359457 | 31392887 | -0.37511131 | 2.12195003 | SNP_A-8596439 | CN_689566 |
| GSM574623_MS2H7 | 1 | 21.1 | 31348374 | 31369498 | -0.41353965 | 2.10864674 | SNP_A-8639864 | SNP_A-1864629 |
| GSM574864_MS7A1 | 1 | 41.6 | 31348374 | 31389948 | -0.33651468 | 2.07441583 | SNP_A-8639864 | CN_689565 |
| GSM574643_MS3B5 | 1 | 17.4 | 31352063 | 31369498 | -0.43796429 | 2.14557806 | CN_689552 | SNP_A-1864629 |
| GSM574776_MS4G6 | 1 | 10.0 | 31359457 | 31369498 | -0.45740622 | 1.94061025 | SNP_A-8596439 | SNP_A-1864629 |
| GSM574938_MS7G6 | 1 | 17.4 | 31352063 | 31369498 | -0.40286481 | 1.97362646 | CN_689552 | SNP_A-1864629 |
| GSM574947_MU1_AP2G12 | 1 | 29.9 | 31352063 | 31381989 | -0.33214267 | 1.84929210 | CN_689552 | SNP_A-8644108 |
| GSM574940_MS7H1 | 1 | 17.4 | 31352063 | 31369498 | -0.35952484 | 1.76130480 | CN_689552 | SNP_A-1864629 |
| GSM574699_MS3G7 | 1 | 40.8 | 31352063 | 31392887 | -0.37106040 | 2.28736993 | CN_689552 | CN_689566 |
| GSM574802_MS5B3 | 1 | 22.2 | 31347287 | 31369498 | -0.34332794 | 1.81672068 | CN_689550 | SNP_A-1864629 |
