# Supplementary material for: A founder deletion in the TRPM1 gene associated with congenital stationary night blindness and myopia is highly prevalent in Ashkenazi Jews
Source: Hum Genome Var. 2019 Sep 12;6:45. doi: 10.1038/s41439-019-0076-4 (PMC6804618; doi:10.1038/s41439-019-0076-4)
Supplement: Supplementary file 9 — Supplementary table 7. [file 41439_2019_76_MOESM9_ESM.docx]

Supplementary Table 7. *TRPM1* deletion associated haplotype prevalence in carriers and non-carriers of Ashkenazi and Non-Ashkenazi origin.

|  | Carrier AJ (n=237) | Carrier Mixed (n=28) | Non-carrier AJ (n=56) | Non-carrier Non-AJ (n=55) |
| --- | --- | --- | --- | --- |
| No. samples with founder haplotype consistent genotypes | 237 | 28 | 25 | 29 |
| No. samples with founder haplotype inconsistent genotypes | 0 | 0 | 31 | 26 |
