# Supplementary material for: A founder deletion in the TRPM1 gene associated with congenital stationary night blindness and myopia is highly prevalent in Ashkenazi Jews
Source: Hum Genome Var. 2019 Sep 12;6:45. doi: 10.1038/s41439-019-0076-4 (PMC6804618; doi:10.1038/s41439-019-0076-4)
Supplement: Supplementary file 10 — Supplementary figs. legend. [file 41439_2019_76_MOESM10_ESM.docx]

**Figure S1. Size and genomic distribution of deletions detected by the Affymetrix 6.0 array.** Stylized visualization of the *TRPM1* gene from Genotyping Console shows exons as blue bars. Red lines represent heterozygous deletion regions as detected by the Canary Algorithm. Black lines represent homozygous deletion regions in one proband each from Families 1 and 2.

**Figure S2.** **Carrier frequency by country of origin.** Bar chart shows carrier frequencies for individuals classified based on their grandparental ancestry. The numbers above each bar represent the frequency as a percentage. Numbers below the bars show the actual number of samples per country.
